# Plano de Ação de Pesquisa Clínica no Brasil: análise do processo de implementação e principais resultados, 2018-2025

**DOI:** 10.1590/0102-311XPT240525

**Published:** 2026-07-27

**Authors:** Giovanny Vinícius Araújo de França, Evandro de Oliveira Lupatini, Michelle Zanon Pereira, Raisa Breda Tôso Sfalsini, Andréa Leite Ribeiro, Karla Andreia Mette Waldrich, Jaqueline Chueke Pureza, João Paulo Alves Oliveira, Patrícia de Souza Boaventura, Luciana Hentzy Moraes, Meiruze Sousa Freitas, Fernanda De Negri

**Affiliations:** 1 Secretaria de Ciência, Tecnologia e Inovação em Saúde, Ministério da Saúde, Brasília, Brasil.

**Keywords:** Estudo Clínico, Política Nacional de Ciência, Tecnologia e Inovação, Sistema Único de Saúde, Clinical Study, National Science, Technology and Innovation Policy, Unified Health System, Estudio Clínico, Política Nacional de Ciencia, Tecnología e Innovación, Sistema Único de Salud

## Abstract

Este artigo apresenta uma avaliação do Plano de Ação de Pesquisa Clínica no Brasil (PAPCB), instituído pelo Ministério da Saúde em 2018. Trata-se de um estudo de avaliação de política pública *ex post*, com base em análise documental referente ao período entre 2018 e 2025. Foram considerados os seis eixos estratégicos do PAPCB: regulação ética, regulação sanitária, fomento científico e tecnológico, formação em pesquisa clínica, Rede Nacional de Pesquisa Clínica (RNPC) e gestão do conhecimento. Os resultados demonstram avanços expressivos no fomento científico e na capacitação profissional, com ampla execução das ações previstas. No eixo de formação, ressalta-se a qualificação de mais de 45 mil profissionais. A RNPC passou por reestruturação, dando origem à Rede Brasileira de Pesquisa Clínica (RBPClin). Persistem, contudo, desafios estruturais, como a necessidade de modernização da Plataforma Brasil, o aprimoramento da gestão do conhecimento e o fortalecimento da colaboração em rede. Recomenda-se a revisão e a atualização do PAPCB, incorporando estratégias voltadas à superação dessas lacunas, de modo a consolidar a capacidade científica, tecnológica e institucional do país em pesquisa clínica.

## Abstract (en)

This study evaluated the Clinical Research Action Plan in Brazil (PAPCB, acronym in Portuguese), established in 2018 by the Brazilian Ministry of Health. This ex post public policy evaluation study was based on documentary analysis for the 2018-2025 period. The six strategic axes of the PAPCB were considered: ethical regulation, sanitary regulation, scientific and technological promotion, training in clinical research, the Clinical Research National Network (RNPC, acronym in Portuguese), and knowledge management. The results showed significant advances in scientific promotion and professional training, with broad execution of the planned actions. The training of more than 45,000 professionals stands out in the training axis. The RNPC underwent restructuring, giving rise to the Brazilian Network of Clinical Research (RBPClin, acronym in Portuguese). However, structural challenges persist, such as the need to modernize Plataforma Brasil, improve knowledge management, and strengthen network collaboration. This study recommends the review and update of the PAPCB, incorporating strategies to overcome these gaps to consolidate the Brazilian scientific, technological, and institutional capacity in clinical research.

## Resumen

Este artículo presenta una evaluación del Plan de Acción de Investigación Clínica en Brasil (PAPCB, por su sigla en portugués), establecido por el Ministerio de Salud en el 2018. Se trata de un estudio de evaluación de política pública *a posteriori*, basado en el análisis de documentos que abarcan el período comprendido entre el 2018 y el 2025. Se consideraron los seis ejes estratégicos de la PAPCB: regulación ética, regulación sanitaria, fomento científico y tecnológico, formación en investigación clínica, Red Nacional de Investigación Clínica (RNPC, por su sigla en portugués) y gestión del conocimiento. Los resultados demuestran un progreso significativo en el fomento científico y en la capacitación profesional, con una amplia implementación de las acciones planificadas. En el eje de la formación, destaca la cualificación de más de 45.000 profesionales. La RNPC se reestructuró, dando lugar a la Red Brasileña de Investigación Clínica (RBPClin, por su sigla en portugués). Sin embargo, persisten desafíos estructurales, como la necesidad de modernizar la Plataforma Brasil, mejorar la gestión del conocimiento y fortalecer la colaboración en red. Se recomienda revisar y actualizar el PAPCB, incorporando estrategias destinadas a superar estas deficiencias, con el fin de consolidar la capacidad científica, tecnológica e institucional del país en investigación clínica.

## Introdução

A pesquisa clínica constitui um componente central do desenvolvimento científico em saúde, contribuindo para a geração de evidências que orientam políticas públicas, a incorporação de tecnologias e o aprimoramento da atenção à saúde ^1^. No Brasil, nação que abriga o maior sistema de saúde público e universal do mundo, o Sistema Único de Saúde (SUS), fomentar um ecossistema de pesquisa autossuficiente e inovador transcende o desenvolvimento científico, tornando-se uma questão de soberania econômica e sanitária ^2^. Nesse contexto, o Ministério da Saúde, por meio de sua Secretaria de Ciência, Tecnologia e Inovação em Saúde (SCTIE/MS), tem atuado de forma sistemática para cultivar um ambiente propício à pesquisa de alto impacto, intrinsecamente alinhada às necessidades de saúde do país.

Esse compromisso foi formalizado e estruturado por meio do Plano de Ação de Pesquisa Clínica no Brasil (PAPCB) ^3^, instituído pela *Portaria GM/MS nº 559/2018*. O PAPCB foi concebido não apenas como um conjunto de diretrizes, mas como um instrumento dinâmico de planejamento e gestão, projetado para organizar prioridades, direcionar investimentos públicos e, crucialmente, fomentar a colaboração entre os diversos atores do cenário de ciência e inovação do Brasil, incluindo governo, academia, setor produtivo e sociedade civil.

Historicamente, o Brasil enfrenta uma significativa dependência de tecnologias de saúde estrangeiras ^4^. O Plano de Ação para a Neoindustrialização (2024-2026) ilustra essa vulnerabilidade de forma contundente, relatando que a produção nacional atende a apenas 42% das necessidades de saúde do país, enquanto as importações de insumos básicos chegam a aproximadamente 90%, a um custo anual estimado de US$ 20 bilhões ^5^. Essa dependência não apenas sobrecarrega as finanças públicas, mas também limita a capacidade do país de responder a emergências sanitárias e de endereçar doenças prevalentes em sua própria população, com suas particularidades genéticas e epidemiológicas.

Para alcançar seu objetivo central de ampliar a capacidade do Brasil de desenvolver e atrair pesquisas clínicas de excelência internacional, o PAPCB foi estruturado em seis eixos estratégicos integrados: regulação ética, regulação sanitária, fomento científico e tecnológico, formação em pesquisa clínica, Rede Nacional de Pesquisa Clínica (RNPC) e gestão do conhecimento. Sua elaboração contou com a participação de diversos atores estratégicos do setor, sendo que cada eixo aborda um componente essencial do ecossistema de pesquisa.

Apesar da relevância do PAPCB, não foram instituídos mecanismos formais de monitoramento e avaliação no momento de sua elaboração, o que dificultou a mensuração de seus resultados em tempo real e o acompanhamento sistemático de sua implementação. Em países como o Reino Unido e os Estados Unidos, existem políticas nacionais consolidadas e sistemas estruturados de avaliação periódica dos ecossistemas de pesquisa clínica ^6^ ^,^ ^7^. Em contraste, em nações de renda média, como o Brasil, onde desigualdades regionais, limitações orçamentárias e fragilidades regulatórias são mais acentuadas, observa-se escassez de avaliações sistemáticas dessas políticas, exigindo abordagens adaptadas, integradas e sustentáveis ^8^ ^,^ ^9^.

Este artigo objetivou analisar a implementação do PAPCB, no período de 2018 a 2025, identificando os avanços e os desafios que persistem para o fortalecimento da pesquisa clínica no Brasil.

## Método

**Figura 1:**
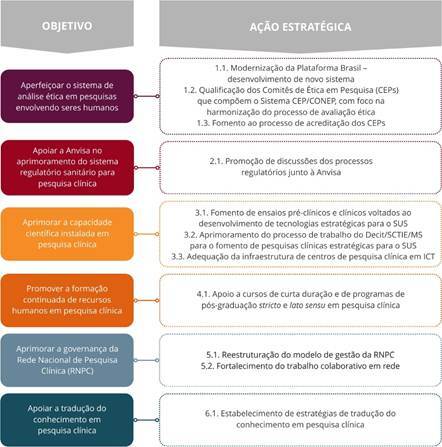
Objetivos e ações estratégicas definidos no âmbito do Plano de Ação de Pesquisa Clínica no Brasil, 2018 a 2025.

Trata-se de um estudo de avaliação de política pública *ex post*, com foco na verificação dos resultados alcançados durante a implementação do PAPCB, desde a sua institucionalização em 2018 até outubro de 2025. O PAPCB publicado foi estruturado da seguinte forma: diagnóstico situacional, objetivos e para cada um dos eixos foram estabelecidas ações estratégicas e atividades. Buscou-se examinar em que medida os objetivos e ações estratégicas definidos pelo PAPCB (Figura 1) foram alcançados, com base em uma avaliação interna, conduzida por integrantes da equipe do Departamento de Ciência e Tecnologia (Decit/SCTIE/MS), especialmente por colaboradores da Coordenação-Geral de Ações Estratégicas em Pesquisa Clínica (CGPClin/Decit/SCTIE/MS).

Anvisa: Agência Nacional de Vigilância Sanitária; CONEP: Comissão Nacional de Ética em Pesquisa; Decit/SCTIE/MS: Departamento de Ciência e Tecnologia/Secretaria de Ciência, Tecnologia e Inovação em Saúde/Ministério da Saúde; ICT: instituições científicas e tecnológicas; SUS: Sistema Único de Saúde.

Para isso, realizou-se uma análise documental abrangente, incluindo: portarias publicadas, parcerias firmadas por meio de termo de execução descentralizada (TED), editais, ofícios, relatórios técnicos do Decit/SCTIE/MS e demais registros administrativos do Sistema Eletrônico de Informações (SEI) do governo brasileiro, divulgações públicas da Agência Nacional de Vigilância Sanitária (Anvisa) e documentos oficiais do Conselho Nacional de Saúde (CNS) e da Comissão Nacional de Ética em Pesquisa (CONEP).

A estrutura analítica deste artigo espelha a construção do PAPCB, organizando os achados de acordo com os seis eixos estratégicos definidos no Plano. Para cada eixo, as ações implementadas, os resultados alcançados e os desafios identificados foram sistematicamente extraídos, sintetizados e contextualizados.

Para avaliar o progresso de cada ação estratégica foi estabelecida uma métrica quantitativa, atribuindo uma porcentagem de execução em uma escala de 0% a 100% para cada ação estratégica, baseando-se no *status* de cada uma das atividades previstas em cada ação. Três avaliadores (G.V.A.F., M.Z.P. e R.B.T.S.) atribuíram, de forma independente, um percentual de execução para cada ação estratégica. Posteriormente, os valores atribuídos foram discutidos e consensuados entre os avaliadores, resultando em um percentual final para cada ação. Para facilitar a interpretação dos resultados, foi adotada uma classificação qualitativa em três grupos:

Insatisfatório (0%-50%): a ação não foi executada ou não apresentou progresso substancial.

Alerta (50%-74%): a ação foi iniciada e apresenta resultados parciais, mas com progresso limitado em relação às suas metas.

Satisfatório (75%-100%): a ação foi totalmente concluída ou apresenta resultados substanciais.

A definição desses intervalos percentuais teve caráter operacional, sendo estabelecida por consenso entre os avaliadores para facilitar a interpretação do grau de implementação das ações estratégicas do PAPCB. Embora esses limiares não derivem diretamente de um referencial normativo específico, classificações percentuais semelhantes são frequentemente empregadas em avaliações administrativas de programas governamentais para distinguir níveis de execução das atividades planejadas. Assim, os intervalos utilizados devem ser interpretados como parâmetros analíticos convencionais para fins de monitoramento da implementação das ações do PAPCB.

Essa abordagem de métodos mistos permite uma avaliação integrada e baseada em evidências da execução do PAPCB. Os achados foram submetidos a um processo de validação envolvendo gestores e técnicos do Ministério da Saúde, diretamente engajados na implementação do PAPCB, de modo a conferir maior precisão e relevância prática para as conclusões apresentadas.

Este estudo utilizou exclusivamente dados secundários, consolidados e/ou de acesso público, não envolvendo a participação direta ou indireta de seres humanos. Dessa forma, conforme dispõem a *Resolução nº 674/2022* do CNS e a *Lei nº 14.874/2024*, não foi necessária a submissão do projeto à apreciação ética de um comitê de ética em pesquisa (CEP).

## Resultados

**Figura 2:**
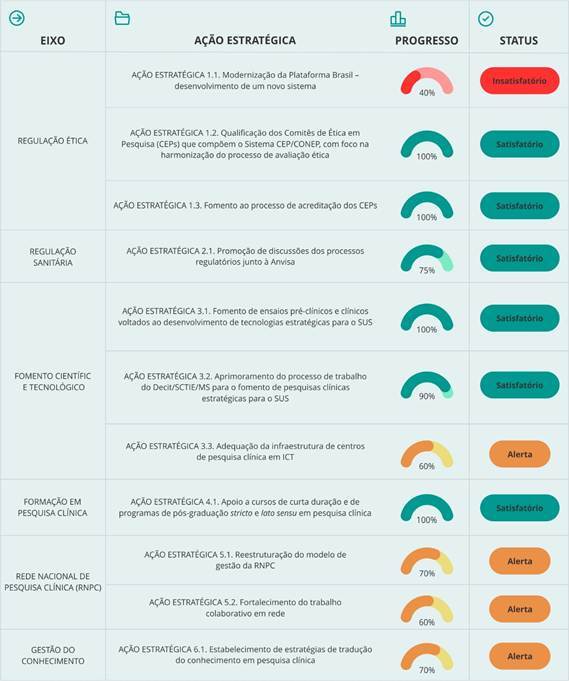
Progresso e *status* das ações estratégicas do Plano de Ação de Pesquisa Clínica no Brasil, 2018 a 2025.

A Figura 2 apresenta o progresso e *status* das ações estratégicas distribuídas entre seis eixos temáticos do PAPCB. Observa-se que a maioria das ações alcançou desempenho satisfatório, especialmente nos eixos de regulação ética, regulação sanitária, fomento científico e tecnológico, e formação em pesquisa clínica. Em contraste, algumas ações apresentam alertas ou desempenho insatisfatório, indicando desafios específicos na modernização da Plataforma Brasil (40%), na adequação da infraestrutura de centros de pesquisa clínica (60%), na reestruturação do modelo de gestão da RNPC (70%), no fortalecimento do trabalho colaborativo em rede (60%) e na tradução do conhecimento (70%) (Tabela 1). A seguir, são apresentados os principais resultados referentes a cada um dos eixos temáticos do PAPCB.

Anvisa: Agência Nacional de Vigilância Sanitária; CONEP: Comissão Nacional de Ética em Pesquisa; Decit/SCTIE/MS: Departamento de Ciência e Tecnologia/Secretaria de Ciência, Tecnologia e Inovação em Saúde/Ministério da Saúde; ICT: instituições científicas e tecnológicas; SUS: Sistema Único de Saúde.

**Tabela 1:** Avaliação quantitativa do progresso das ações estratégicas do Plano de Ação de Pesquisa Clínica no Brasil, 2018 a 2025.

Eixo estratégico/Ação estratégica	Progresso (%)	Classificação	Justificativa da classificação (com base em marcos)
1. Regulação ética			
1.1. Modernização da Plataforma Brasil	40%	Insatisfatório	Apenas a fase de planejamento (consulta pública) foi concluída. O desenvolvimento do sistema está em andamento parcial e as etapas críticas de teste piloto e monitoramento não foram iniciadas, comprometendo a entrega final
1.2. Qualificação do Sistema CEP/CONEP	100%	Satisfatório	O ciclo completo foi executado: o projeto Q-CEP realizou visitas e treinou 11.197 pessoas; 15 cursos EaD foram criados e migrados para uma plataforma pública permanente (UNA-SUS), garantindo a sustentabilidade da ação
1.3. Fomento à acreditação dos CEPs	100%	Satisfatório	A ação atingiu todos os seus marcos: discussões e consulta pública levaram à publicação da *Resolução nº 674/2022* do CNS; chamadas públicas foram lançadas, CEPs foram selecionados e certificados, e o monitoramento foi implementado
2. Regulação sanitária			
2.1. Discussões com a Anvisa	75%	Satisfatório	Atingiu a maior parte dos objetivos com a realização contínua de eventos, reuniões técnicas e colaborações para harmonizar normas éticas e sanitárias, demonstrando uma interlocução entre os órgãos
3. Fomento científico e tecnológico			
3.1. Fomento de ensaios pré-clínicos e clínicos	100%	Satisfatório	O ciclo de fomento foi integralmente cumprido com a realização de prospecção, o lançamento de múltiplas chamadas públicas estratégicas (COVID-19, vacinas, saúde de precisão), a contratação direta de estudos de interesse do SUS, o monitoramento e a avaliação desses projetos
3.2. Aprimoramento do processo do Decit/SCTIE/MS	90%	Satisfatório	Atingiu seus marcos principais ao redefinir e institucionalizar processos internos de seleção e monitoramento por meio de manuais, POPs e comitês de governança. As parcerias público-privadas foram estimuladas com sucesso, embora ainda em consolidação
3.3. Adequação da infraestrutura de centros	60%	Alerta	A ação avançou nas fases iniciais e cruciais de diagnóstico com a contratação de projetos para mapear os centros de pesquisa e aprimorar o ReBEC. Contudo, a fase principal de incentivo e modernização da infraestrutura ainda não foi implementada, aguardando esses resultados
4. Formação em pesquisa clínica			
4.1. Apoio a cursos e pós-graduação	100%	Satisfatório	Atingiu plenamente seus objetivos com a oferta de um portfólio concreto de cursos (introdutório, intermediário, especialização e mentoria) por meio do PROADI-SUS, certificando dezenas de milhares de profissionais e garantindo a continuidade das formações
5. Rede Nacional de Pesquisa Clínica (RNPC)			
5.1. Reestruturação do modelo de gestão	70%	Alerta	O ciclo completo de reestruturação foi concluído: o modelo foi diagnosticado, desenhado em parceria com *stakeholders*, formalizado pelo *Decreto nº 11.287/2022* e amplamente divulgado em diversos fóruns estratégicos
5.2. Fortalecimento do trabalho colaborativo	60%	Alerta	Os resultados são parciais. Houve sucesso em ações pontuais de cooperação internacional (ensaios da OMS), mas a base para a colaboração sistêmica - um ambiente virtual para divulgar competências - está em fase muito inicial, limitando a integração da rede
6. Gestão do conhecimento			
6.1. Estratégias de tradução do conhecimento	70%	Alerta	A ação foi iniciada com entregas importantes, como o apoio ao ReBEC e a realização de eventos. No entanto, o relatório aponta uma falha estratégica na divulgação do próprio plano de ação e a falta de um canal de comunicação estruturado, indicando que os resultados são mais pontuais do que sistêmicos

Anvisa: Agência Nacional de Vigilância Sanitária; CEP: comitê de ética em pesquisa; CNS: Conselho Nacional de Saúde; CONEP: Comissão Nacional de Ética em Pesquisa; Decit/SCTIE/MS: Departamento de Ciência e Tecnologia/Secretaria de Ciência, Tecnologia e Inovação em Saúde/Ministério da Saúde; EaD: educação a distância; OMS: Organização Mundial da Saúde; POP: procedimento operacional padrão; PROADI-SUS: Programa de Apoio ao Desenvolvimento Institucional do Sistema Único de Saúde; Q-CEP: *Qualificação dos Comitês de Ética em Pesquisa*; ReBEC: Registro Brasileiro de Ensaios Clínicos; SUS: Sistema Único de Saúde; UNA-SUS: Universidade Aberta do SUS.

### Eixo 1: Regulação ética

Em conformidade com a *Resolução nº 506/2016* do CNS e o disposto no PAPCB, um avanço foi a acreditação de CEPs no Estado de São Paulo, por meio de editais lançados em 2020 e 2022, que resultaram na acreditação de oito CEPs até o ano de 2024. No entanto, ainda se observa a necessidade de ampliar esse processo para as demais regiões do país, de modo a promover maior equidade territorial na consolidação do sistema de ética em pesquisa. Durante a implementação das ações de acreditação, e com base nas necessidades identificadas pelos CEPs envolvidos, foram elaborados diversos materiais educativos de apoio, incluindo a série de vídeos *Diálogo em Pílulas*, o manual *Construção do Parecer Consubstanciado - Sugestões de Padronização* e o *Banco de Pendências*. Todos esses recursos foram desenvolvidos para auxiliar e fortalecer o trabalho dos membros dos CEPs.

Outra estratégia implementada foi o projeto *Qualificação dos Comitês de Ética em Pesquisa (Q-CEP)*, executado em parceria com a Associação Hospitalar Moinhos de Vento (AHMV; Porto Alegre, Rio Grande do Sul) via Programa de Apoio ao Desenvolvimento Institucional do Sistema Único de Saúde (PROADI-SUS). Entre 2019 e 2021, foram realizadas visitas de treinamento a 832 CEPs, sendo 259 presenciais e 573 remotas, cobrindo quase a totalidade dos comitês do país e capacitando diretamente 11.197 profissionais. Durante essas visitas, foram elaborados planos de ação individualizados e colaborativos com metas de curto, médio e longo prazos para aprimorar os processos de cada comitê. Além disso, o projeto criou 15 cursos online, gratuitos, autoinstrucionais e de livre acesso, abordando temas como consentimento livre e esclarecido, procedimentos da Plataforma Brasil e marcos regulatórios. Esses cursos registraram 48.391 inscrições e emitiram 28.514 certificados, contribuindo de forma significativa para o aprimoramento do conhecimento ético em todo o sistema ^10^.

Apesar dos avanços alcançados, a modernização da Plataforma Brasil permanece como um gargalo crítico e o principal ponto de vulnerabilidade identificado. O desenvolvimento de um novo sistema, mais eficiente e flexível, avançou lentamente, apresentando desempenho insatisfatório conforme os critérios de avaliação. O Q-CEP foi suspenso temporariamente em 2023, em razão de reestruturações na gestão de contratos de tecnologia da informação no âmbito do Ministério da Saúde e, embora tenha sido retomado em 2024, até setembro de 2025 ainda não havia entrado nas fases de teste piloto ou monitoramento com usuários. Esse atraso compromete a agilidade e a eficiência de todo o processo de revisão ética no Brasil, gerando sobrecarga para o Sistema Nacional de Ética em Pesquisa com Seres Humanos (SINEP).

Em 7 de outubro de 2025, foi publicado o *Decreto nº 12.651*, que regulamenta a *Lei nº 14.874/2024* e institui o SINEP, constituindo um marco regulatório para o aprimoramento do sistema brasileiro de regulação ética. Essa atualização normativa visa a fortalecer a capacidade do Estado de assegurar celeridade, transparência e qualidade nas análises de protocolos de pesquisa, preservando os princípios éticos fundamentais que orientam a proteção, integridade e o bem-estar dos participantes de pesquisa. Além disso, cria plataforma de pesquisas com seres humanos, que deverá ser o sistema eletrônico integrado de cadastro, protocolo, informação e análise de pesquisas, sob a governança do Ministério da Saúde.

### Eixo 2: Regulação sanitária

Focado em apoiar a Anvisa em seus esforços para aprimorar processos regulatórios, contribuindo para maior eficiência, transparência e alinhamento com boas práticas internacionais, este eixo alcançou uma execução satisfatória. Isso reflete uma interação contínua entre o Ministério da Saúde e a autoridade sanitária nacional. As principais atividades incluíram a promoção de um diálogo entre o setor regulado (pesquisadores, indústria) e os reguladores. Foram realizados eventos, como o *I Fórum de Biobancos*, o *I Fórum Internacional de Pesquisa Clínica*, três edições do *Summit Internacional sobre Saúde de Precisão* e seminários de monitoramento das pesquisas contratadas por meio das chamadas públicas de fomento à pesquisa científica. Essa colaboração foi crucial para alinhar os objetivos científicos com as exigências regulatórias desde o início dos projetos.

Ademais, houve um esforço conjunto para promover a harmonização entre as diretrizes sanitárias da Anvisa e as normas éticas da CONEP. Reuniões periódicas entre as duas instâncias foram estabelecidas, especialmente durante a pandemia de COVID-19, para alinhar procedimentos de pesquisas com amostras biológicas humanas e dados sensíveis (subsidiando a *Resolução nº 771/2025* do CNS). A participação do Brasil como membro do International Council for Harmonisation of Technical Requirements for Pharmaceuticals for Human Use (ICH; Conselho Internacional para a Harmonização de Requisitos Técnicos para Produtos Farmacêuticos de Uso Humano), desde 2016, também foi fundamental, levando à adoção de modelos internacionais harmonizados para a submissão de *Dossiês de Desenvolvimento Clínico de Medicamento* (DDCM), o que aumentou a previsibilidade e a eficiência.

Apesar dos avanços observados nesse eixo entre 2018 e 2025, permanece a necessidade de fortalecer a articulação entre o Ministério da Saúde e a Anvisa, de forma a promover uma harmonização efetiva entre os processos de regulação ética e sanitária na pesquisa clínica. Essa integração é fundamental para reduzir sobreposições e tempos de análise, assegurar maior previsibilidade regulatória e favorecer o ambiente de inovação e desenvolvimento de estudos clínicos no país.

### Eixo 3: Fomento científico e tecnológico

Este eixo foi projetado para fortalecer a capacidade científica do Brasil por meio do financiamento de pesquisas estratégicas, da promoção de parcerias público-privadas e do aprimoramento da infraestrutura dos centros de pesquisa. De modo geral, este foi o eixo de maior sucesso do PAPCB, com resultados concretos e expressivos.

O destaque dessa estratégia foi o lançamento de numerosas Chamadas Públicas em parceria com o Conselho Nacional de Desenvolvimento Científico e Tecnológico (CNPq) (Tabela 2). Além disso, foi realizada a contratação direta de projetos estratégicos, incluindo estudos sobre vacinas (p.ex.: dengue, febre amarela, reforços para COVID-19), doenças negligenciadas (p.ex.: doença de Chagas), oncologia e terapias avançadas, como a terapia com células CAR-T desenvolvida no Instituto Nacional de Câncer (INCA).

**Tabela 2:** Principais chamadas públicas de fomento à pesquisa realizadas no âmbito do Plano de Ação de Pesquisa Clínica no Brasil, 2018 a 2025.

Ano	Chamada	Tema principal	Projetos financiados
2018	MS-SCTIE-Decit/CNPq nº 12/2018	Pesquisas de inovação em saúde	18
2018	CNPq/MS/SCTIE/Decit nº 19/2018	Fitoterápicos	12
2018	CNPq/MS-SCTIE-Decit nº 01/2018	Pesquisas em resistência aos antimicrobianos	13
2019	CNPq/MS-SCTIE-Decit nº 22/2019	Doenças transmissíveis e negligenciadas	15
2020	MCTI/CNPq/FNDCT/MS/SCTIE/Decit nº 07/2020	Enfrentamento da COVID-19 e síndromes respiratórias	117
2022	CNPq/Decit/SCTIE/MS nº 49/2022	Pesquisas estratégicas com vacinas nacionais	15
2023	CNPq/Decit/SCTIE/MS nº 16/2023	Saúde de precisão	95
2023	CNPq/Decit/SCTIE/MS nº 21/2023	Estudos transdisciplinares em saúde coletiva	5
2024	CNPq/Decit/SCTIE/MS nº 32/2024	Pesquisas pré-clínicas e clínicas	50
2024	CNPq/Decit/SCTIE/MS nº 33/2024	Genômica e saúde pública de precisão	56
Total			396

Os esforços para incentivar parcerias público-privadas também progrediram significativamente, alcançando 70% de execução. Um marco importante nesse processo foi o lançamento, em 2022, de uma chamada pública em parceria com a Empresa Brasileira de Pesquisa e Inovação Industrial (EMBRAPII) para a criação de um Centro de Competência em Terapias Avançadas, na qual o Hospital Albert Einstein (São Paulo) foi selecionado. Esse centro foi projetado para cofinanciar projetos de inovação com empresas, conectando a pesquisa acadêmica ao desenvolvimento industrial.

O fomento a ensaios clínicos estratégicos para o SUS foi ampliado por meio dos programas de renúncia fiscal e imunidade tributária: PROADI-SUS, Programa Nacional de Apoio à Atenção Oncológica (Pronon) e o Programa Nacional de Apoio à Atenção da Saúde da Pessoa com Deficiência (Pronas/PCD). Tais programas se consolidaram como instrumentos relevantes de apoio à pesquisa aplicada. Entre 2018 e 2025, o estímulo à submissão de projetos alinhados ao PAPCB resultou na aprovação de 87 ensaios clínicos, totalizando um investimento de R$ 672,2 milhões. Essas iniciativas fortaleceram a capacidade nacional de condução de estudos clínicos em áreas prioritárias para o SUS, como doenças crônicas e infecciosas, e promoveram inovações em campos emergentes, como inteligência artificial, transplantes e malformações congênitas, contribuindo para o avanço científico e a incorporação de tecnologias em saúde mais eficazes e sustentáveis.

Observou-se que os avanços na modernização e estruturação dos centros de pesquisa clínica no país foram limitados. A adequação da infraestrutura desses centros ocorreu de forma desigual ao longo do período estudado, refletindo disparidades regionais e institucionais. Ressalta-se, ainda, a inexistência de um levantamento nacional preciso e atualizado sobre o número, a distribuição geográfica e o grau de maturidade dos centros de pesquisa clínica, o que dificulta o planejamento estratégico, a alocação de recursos e a definição de políticas voltadas ao fortalecimento da capacidade instalada no país.

Nesse contexto, o Decit/SCTIE/MS apoiou duas iniciativas estratégicas: o *TED nº 19/2024*, firmado com o CNPq, e que permitiu encomendar à Universidade Estadual Paulista (UNESP) de Botucatu (São Paulo) a realização do projeto *Desenvolvimento e Execução do Mapeamento e Diagnóstico Situacional dos Centros de Pesquisa Clínica no Brasil*, cujo objetivo é gerar uma base de dados atualizada sobre capacidade instalada; e o *TED nº 133/2023*, firmado com a Fundação Oswaldo Cruz (FIOCRUZ), para a *Criação, Expansão e Aperfeiçoamento de Funcionalidades do Registro Brasileiro de Ensaios Clínicos (ReBEC)*, incluindo a incorporação de um módulo específico para cadastro de centros. Essas ações visam a estruturar um sistema nacional de informação em pesquisa clínica, essencial para o planejamento, o fomento direcionado e a promoção da transparência no ecossistema de pesquisa brasileiro.

Outra ação com enfoque no aprimoramento do processo de trabalho do Decit/SCTIE/MS se refere à publicação da *Portaria nº 4.282/2022* do Ministério da Saúde. Essa Portaria institui o manual instrutivo para o financiamento de projetos de pesquisa, detalhando as melhores práticas e orientações para submissão, bem como os procedimentos para a disseminação dos resultados científicos.

### Eixo 4: Formação em pesquisa clínica

Enfrentar o déficit de profissionais qualificados foi uma prioridade, e este eixo alcançou uma execução satisfatória. A estratégia focou no apoio a uma gama de programas educacionais, desde cursos de curta duração até pós-graduações. Um modelo de sucesso foi a parceria com Hospitais de Excelência por meio do PROADI-SUS. Entre 2019 e 2025, instituições como o Hospital Alemão Oswaldo Cruz (HAOC; São Paulo) e o AHMV ofereceram um conjunto de cursos que capacitaram milhares de profissionais (Tabela 3). Dentre eles, destacam-se:

**Tabela 3:** Cursos de formação em pesquisa clínica oferecidos pelo Programa de Apoio ao Desenvolvimento Institucional do Sistema Único de Saúde (PROADI-SUS) no âmbito do Plano de Ação de Pesquisa Clínica no Brasil, 2018 a 2025.

Instituição	Tipo de formação/curso	Período de referência	Profissionais certificados/Vagas
HAOC	Curso introdutório de pesquisa clínica	2019-2023	5.276 certificados
HAOC	Curso intermediário de pesquisa clínica	2019-2023	2.866 certificados
HAOC	Curso de especialização *lato sensu* em pesquisa clínica	2019-2023	136 certificados
HAOC	Programa de mentoria em pesquisa clínica	2024-2025	30 vagas ofertadas
AHMV	Curso de gestão da qualidade em pesquisa clínica	2019-2021	128 certificados
AHMV	Cursos autoinstrucionais (14 módulos)	2020-2025	36.665 certificados
Total			45.071 profissionais certificados

AHMV: Associação Hospitalar Moinhos de Vento; HAOC: Hospital Alemão Oswaldo Cruz.

O HAOC ofereceu cursos em níveis distintos (introdutório, intermediário e especialização *lato sensu*), certificando mais de 8 mil profissionais em diversas edições. Também lançou um programa de mentoria para centros de pesquisa em 2024.

O AHMV ofereceu um curso de *Gestão da Qualidade em Pesquisa Clínica* e desenvolveu 14 módulos autoinstrucionais online sobre temas que vão desde o desenho de protocolos até a gestão de dados. Esses módulos, somando as ofertas na plataforma do hospital e na plataforma pública da Universidade Aberta do SUS (UNA-SUS; Brasília, Distrito Federal), certificaram mais de 36 mil profissionais.

Para garantir a acessibilidade a longo prazo, o Ministério da Saúde viabilizou a migração desses cursos autoinstrucionais para a UNA-SUS, onde permanecem disponíveis gratuitamente.

### Eixo 5: Rede Nacional de Pesquisa Clínica (RNPC)

Este eixo visou a reestruturar a governança da RNPC para torná-la mais concreta, participativa e eficiente. A reestruturação do modelo de gestão da RNPC alcançou uma execução satisfatória. Inicialmente, foi atualizado o diagnóstico do cenário da pesquisa clínica no país, incluindo consultas a órgãos governamentais, como os Ministérios da Educação e da Ciência, Tecnologia e Inovação, entidades responsáveis pela regulação ética e sanitária (Anvisa e CONEP), instituições de pesquisa, representantes da indústria, associações de pacientes, entre outros atores envolvidos na pesquisa clínica.

Esse processo de diálogo e participação resultou na criação formal da Rede Brasileira de Pesquisa Clínica (RBPClin), instituída pelo *Decreto nº 11.287/2022*. Apesar da publicação do Decreto, a rede ainda se encontra em processo de composição, não tendo sido formalizado o seu conselho deliberativo, o que evidencia a necessidade de avanços na consolidação de sua governança e operacionalização plena.

Contudo, permanece uma lacuna significativa no fortalecimento do trabalho colaborativo em rede, o que ficou evidente na execução de apenas 60% da ação e foi classificada com o *status* de alerta. Embora a estrutura de governança esteja estabelecida, persistem desafios para fomentar a integração, a comunicação e a cooperação efetivas entre os centros membros. As iniciativas para criar um ambiente virtual para divulgar as competências dos membros da rede foram iniciadas, mas estão em estágio inicial, com apenas 20% de execução, limitando o potencial de sinergia da rede, embora articulações pontuais, como a indicação de centros brasileiros para estudos da Organização Mundial da Saúde (OMS), tenham sido bem-sucedidas.

### Eixo 6: Gestão do conhecimento

O objetivo deste eixo foi reduzir a lacuna entre a geração de evidências científicas e sua aplicação nos processos de tomada de decisão no âmbito do SUS. Essa área foi identificada como um ponto de fragilidade na execução do PAPCB, apresentando desempenho em nível de alerta, o que evidencia a necessidade de fortalecer os mecanismos de integração entre pesquisa e gestão.

Entre as principais iniciativas desenvolvidas, destaca-se a realização do *I Fórum Internacional de Pesquisa Clínica*, em 2023, que constituiu um marco na promoção do diálogo interinstitucional e na disseminação de boas práticas de gestão do conhecimento. Ressalta-se, também, a atuação do Decit/SCTIE/MS durante a pandemia de COVID-19, por meio do Monitoramento do Desenvolvimento de Vacinas contra a COVID-19, que resultou na publicação de dois relatórios técnicos, em 2021 e 2022. Além disso, os seminários parciais e finais dos projetos contratados via chamadas públicas configuraram importantes oportunidades de tradução e disseminação do conhecimento produzido, especialmente por aproximarem as áreas técnicas do Ministério da Saúde e grupos de pesquisa, favorecendo a incorporação de evidências científicas na formulação de políticas e ações estratégicas.

Contudo, apesar desses progressos, ainda se observa a ausência de uma estratégia sistêmica e articulada para a tradução do conhecimento, capaz de integrar de forma contínua os resultados das pesquisas aos processos decisórios e às políticas públicas em saúde.

Não foram identificados mecanismos institucionais de gestão do conhecimento, capazes de promover o uso estratégico das evidências científicas na formulação, implementação e avaliação de políticas de saúde.

## Discussão

A avaliação da implementação do PAPCB, entre 2018 e 2025, revela progressos importantes, porém marcados desequilíbrios estruturais persistentes. Os maiores sucessos do PAPCB residem em áreas nas quais o investimento direto e a ação programática puderam ser aplicados de forma eficaz como o fomento científico e a formação de recursos humanos.

O direcionamento estratégico de recursos por meio de chamadas públicas alinhou com sucesso a pesquisa às prioridades do SUS, especialmente em resposta à pandemia de COVID-19 e em campos emergentes como a saúde de precisão e as terapias avançadas. Da mesma forma, os programas de capacitação sistemática, em especial via PROADI-SUS, construíram uma massa crítica de profissionais com conhecimento padronizado em pesquisa clínica, um passo fundamental para a melhoria da qualidade e da conformidade regulatória em todo o país.

No entanto, o balanço dos resultados também expõe assimetrias importantes. A mais evidente é o contraste entre o progresso dinâmico no fomento e na formação e a estagnação na modernização tecnológica da Plataforma Brasil. Essa infraestrutura digital é a porta de entrada para todo o sistema de revisão ética, e sua ineficiência cria um gargalo sistêmico que mina muitos dos ganhos em outras áreas. Isso evidencia uma lição fundamental: o avanço de um ecossistema de pesquisa complexo exige progresso sincronizado em todos os seus componentes interdependentes. Sem ferramentas regulatórias modernas e eficientes, o potencial de uma comunidade de pesquisa bem financiada e treinada não pode ser plenamente realizado.

Outro grande desafio é a desigualdade regional. A infraestrutura de pesquisa e o financiamento de projetos permanecem fortemente concentrados na Região Sudeste do Brasil, perpetuando disparidades históricas. Isso não apenas limita o desenvolvimento científico nas regiões Norte e Nordeste, mas também significa que a pesquisa clínica muitas vezes não reflete a vasta diversidade genética e epidemiológica do país, o que configura uma oportunidade perdida para uma nação com uma das populações mais miscigenadas do mundo ^11^ ^,^ ^12^.

Do ponto de vista das políticas de ciência e tecnologia em saúde, a persistência dessas desigualdades territoriais está associada a diferenças históricas na capacidade institucional, disponibilidade de infraestrutura científica e distribuição de recursos humanos qualificados entre as regiões do país. A literatura sobre fortalecimento de sistemas de pesquisa em países de renda média destaca que políticas públicas voltadas à expansão da pesquisa clínica devem incorporar instrumentos específicos para reduzir essas assimetrias, como programas de capacitação institucional, incentivos regionais de financiamento e mecanismos de cooperação interinstitucional entre centros mais consolidados e instituições emergentes. A adoção dessas estratégias pode contribuir para ampliar a participação de regiões historicamente sub-representadas na pesquisa clínica e aumentar a representatividade da diversidade populacional brasileira nos estudos conduzidos no país.

Identificou-se, ainda, uma fragilidade expressiva na gestão do conhecimento. Embora tenha obtido êxito em impulsionar a produção de evidências científicas, o PAPCB não conseguiu estabelecer mecanismos consistentes para a tradução sistemática dessas evidências em políticas e práticas de saúde. Essa lacuna pode ser interpretada à luz da literatura sobre translação do conhecimento em sistemas de saúde, que destaca que a produção de evidências científicas, por si só, não garante a sua incorporação em processos decisórios ou práticas assistenciais ^13^. Para reduzir essa distância entre conhecimento e ação, diversos sistemas de saúde têm desenvolvido mecanismos institucionais voltados à síntese, mediação e disseminação de evidências para gestores e formuladores de políticas, como sínteses orientadas à tomada de decisão, plataformas de evidências e redes de interação entre pesquisadores e tomadores de decisão ^14^ ^,^ ^15^ ^,^ ^16^.

A revisão do PAPCB deve, portanto, priorizar o desenvolvimento de plataformas digitais fundamentais para a gestão e a tradução do conhecimento, bem como a criação de mecanismos de interação contínua entre pesquisadores, gestores e formuladores de políticas, de modo a fortalecer o ciclo de retroalimentação entre evidências e tomada de decisão.

Visando ao aprimoramento do processo de monitoramento e avaliação do PAPCB, destaca-se a necessidade de incorporar métricas específicas de desempenho e impacto, permitindo a mensuração da efetividade das ações implementadas. Além disso, é essencial estabelecer estratégias permanentes de divulgação e transparência sobre o andamento das atividades e os resultados alcançados, promovendo maior responsabilização institucional e facilitando o uso das evidências geradas na gestão pública da saúde.

Este estudo configura-se como uma avaliação de política pública, fundamentada em análise documental e de natureza descritiva e analítica, utilizando os seis eixos estratégicos do PAPCB como arcabouço conceitual para a interpretação dos resultados. A literatura sobre este tipo de estudo distingue diferentes níveis de resultados na avaliação de políticas públicas, diferenciando: insumos (*inputs*), referentes aos recursos mobilizados para a implementação da iniciativa, como financiamento, infraestrutura e recursos humanos; produtos (*outputs*), que correspondem às entregas imediatas das ações executadas, mensuráveis por indicadores operacionais; resultados (*results*), que dizem respeito às mudanças diretas observadas entre os beneficiários ou no contexto da política; e impactos (*impact*), que representam efeitos mais amplos e de longo prazo sobre dimensões sociais, econômicas ou institucionais ^17^ ^,^ ^18^ ^,^ ^19^.

Neste estudo, a análise concentra-se principalmente nos produtos e, em menor medida, em alguns resultados institucionais associados à implementação do PAPCB. Assim, eventuais contribuições mais amplas para o fortalecimento do ecossistema nacional de pesquisa clínica devem ser interpretadas como efeitos potenciais, cuja confirmação exigiria avaliações específicas de impacto.

A adoção de uma metodologia de métodos mistos, que articula métricas quantitativas e classificações qualitativas, permitiu uma apreciação mais abrangente do desempenho institucional e da implementação das ações previstas. Contudo, tal abordagem apresenta limitações, especialmente quanto à validade externa e à generalização dos achados. Parte das metas avaliadas encontrava-se em fase de implementação, o que possivelmente influenciou a interpretação dos resultados e o grau de consolidação das conclusões apresentadas.

A principal limitação metodológica reside na ausência de auditoria externa independente, decorrente do caráter do estudo como uma autoavaliação institucional. A validação dos resultados foi conduzida por gestores e técnicos diretamente envolvidos na execução do PAPCB, o que pode introduzir viés de confirmação e restringir a imparcialidade da análise. Avaliações institucionais conduzidas por equipes diretamente envolvidas na implementação das políticas analisadas apresentam vantagens importantes, como o acesso ampliado a registros administrativos, documentos internos e conhecimento acumulado sobre os processos decisórios e operacionais envolvidos na execução das ações. Entretanto, a literatura de avaliação de políticas públicas reconhece que esse tipo de abordagem pode estar sujeito a vieses de confirmação ou seleção de evidências ^20^. Nesse contexto, os resultados apresentados neste estudo devem ser interpretados principalmente como indicadores administrativos de progresso na implementação das ações do PAPCB, e não como medidas independentes de efetividade ou impacto sistêmico das políticas analisadas.

Apesar dessas restrições, a experiência do PAPCB oferece lições importantes em governança. A reestruturação bem-sucedida da RNPC como RBPClin demonstra o valor de um arcabouço legal claro e de um processo consultivo. No entanto, a dificuldade contínua em fomentar uma colaboração genuína dentro da rede mostra que as estruturas formais são insuficientes se não existirem mecanismos que incentivem o trabalho conjunto. Observa-se também a necessidade de considerar as implicações éticas, legais e sociais em diferentes contextos, bem como implementar políticas de ações afirmativas para promover a equidade racial na pesquisa, lacunas identificadas no plano original.

Os resultados desta avaliação têm contribuído para o processo de revisão e atualização do PAPCB, atualmente em elaboração no âmbito do Ministério da Saúde. A análise das ações implementadas entre 2018 e 2025 fornece subsídios empíricos relevantes para orientar o próximo ciclo estratégico da política, permitindo identificar áreas prioritárias de fortalecimento. Nesse sentido, o presente estudo não apenas analisa retrospectivamente a implementação do PAPCB, mas também contribui para o aperfeiçoamento das futuras estratégias de fortalecimento da pesquisa clínica no país.

## Conclusão

O PAPCB se destaca como uma ação estratégica e, em grande medida, bem-sucedida, que promoveu avanços tangíveis no ecossistema da pesquisa clínica no país. A iniciativa fortaleceu a produção científica por meio de fomento estratégico, ampliou a capacidade de recursos humanos com ações de capacitação em larga escala e aprimorou o diálogo crucial entre os órgãos de regulação ética e sanitária. Essas conquistas estabeleceram uma base mais sólida para que o Brasil conduza pesquisas que sejam, ao mesmo tempo, globalmente competitivas e localmente relevantes para as necessidades do SUS.

Contudo, esta análise aprofundada da implementação do PAPCB expôs deficiências estruturais críticas que devem ser o foco do próximo ciclo estratégico. A modernização da Plataforma Brasil, que deverá ser substituída pela plataforma de pesquisas com seres humanos, é uma prioridade urgente e inegociável. Concomitantemente, uma política nacional deve ser desenvolvida para enfrentar as profundas desigualdades regionais em infraestrutura e fomento à pesquisa. Finalmente, a lacuna entre a geração de conhecimento e a sua aplicação prática deve ser superada por meio de estratégias de tradução do conhecimento dedicadas e sistêmicas. Ao enfrentar suas vulnerabilidades internas e posicionar-se estrategicamente na comunidade científica global, o Brasil poderá alavancar o progresso alcançado pelo PAPCB, avançando em direção a um ecossistema de pesquisa clínica verdadeiramente inovador, sustentável e equitativo, em benefício de toda a sua população.

## Data Availability

As fontes de informação utilizadas no estudo estão indicadas no corpo do artigo.
